# Interleaved configurations of percutaneous epidural stimulation enhanced overground stepping in a person with chronic paraplegia

**DOI:** 10.3389/fnins.2023.1284581

**Published:** 2023-12-07

**Authors:** Ashraf S. Gorgey, Siddharth Venigalla, Muhammad Uzair Rehman, Botros George, Enrico Rejc, Jan J. Gouda

**Affiliations:** ^1^Spinal Cord Injury and Disorders, Richmond VA Medical Center, Richmond, VA, United States; ^2^Department of Physical Medicine and Rehabilitation, Virginia Commonwealth University, Richmond, VA, United States; ^3^ELAGI Center for Physical Therapy and Rehabilitation, Giza, Egypt; ^4^Department of Medicine, University of Udine, Udine, Italy; ^5^Neurosurgery Department, Louran Hospital, Alexandria, Egypt; ^6^Department of Surgery, Wright State University, Dayton, OH, United States

**Keywords:** spinal cord injury, rehabilitation, epidural stimulation, configurations, overground and treadmill walking

## Abstract

Descending motor signals are disrupted after complete spinal cord injury (SCI) resulting in loss of standing and walking. We previously restored standing and trunk control in a person with a T3 complete SCI following implantation of percutaneous spinal cord epidural stimulation (SCES). We, hereby, present a step-by-step procedure on configuring the SCES leads to initiate rhythmic lower limb activation (rhythmic-SCES) resulting in independent overground stepping in parallel bars and using a standard walker. Initially, SCES was examined in supine lying at 2 Hz before initiating stepping-like activity in parallel bars using 20 or 30 Hz; however, single lead configuration (+2, −5) resulted in lower limb adduction and crossing of limbs, impairing the initiation of overground stepping. After 6 months, interleaving the original rhythmic-SCES with an additional configuration (−12, +15) on the opposite lead, resulted in a decrease of the extensive adduction tone and allowed the participant to initiate overground stepping up to 16 consecutive steps. The current paradigm suggests that interleaving two rhythmic-SCES configurations may improve the excitability of the spinal circuitry to better interpret the residual descending supraspinal signals with the ascending proprioceptive inputs, resulting in a stepping-like motor behavior after complete SCI.

## Introduction

Spinal cord epidural stimulation (SCES) facilitated restoration of motor control in persons with complete and incomplete spinal cord injury (SCI) (Harkema et al., 2011; Angeli et al., 2018; Wagner et al., 2018; Gorgey et al., 2020). The use of SCES can transform the dormant lumbosacral segments into active neural circuitry that enhances standing and overground locomotion (Harkema et al., 2011; Wagner et al., 2018). The changes in the lumbosacral neurophysiological status have become apparent via integration of segmental activation, afferent input as well as supraspinal descending controls (Gill et al., 2021; Hachmann et al., 2021). Several published articles described the potential neurophysiological mechanisms that may likely lead to restoration of motor control after SCI. However, the exact mechanisms have yet to be determined (Taccola et al., 2020; Gill et al., 2021; Hachmann et al., 2021).

Implanted electrode arrays were temporospatial used to stimulate the lumbosacral segments and result in establishing motor recruitment curves of the paralyzed knee extensor and ankle muscles (Sayenko et al., 2014; Angeli et al., 2023). Selective recruitment of the rostral and caudal areas of the lumbosacral segments were noted when the narrow and wide spatial SCES configurations were applied (Angeli et al., 2023). Additionally, the authors noted wide variability in the motor evoked response between supine and standing positions for different muscle groups (Angeli et al., 2023). These earlier findings have been effectively translated to perform spatiotemporal mapping of the lumbosacral segment to determine the effective stimulation parameters required to achieve standing (Rejc et al., 2017). The use of single percutaneous lead was used to temporarily activate the posterior root muscle reflex of the spinal circuits and elicited rhythmic flexion/extension movements (Minassian et al., 2004). These findings suggest that neuroprotheses with a single percutaneous lead may be used to activate the lumbosacral segments to achieve synergistic activation patterns and functional movements (Minassian and Hofstoetter, 2016).

The aforementioned findings paved the road to utilize percutaneous SCES to enhance motor recovery in persons with SCI (Gorgey and Gouda, 2022; Gorgey et al., 2023a,b). We noted that a single lead placed at T11-LI may facilitate motor recovery in a T3 AIS A person with a chronic complete SCI (Gorgey and Gouda, 2022). The person was able to achieve independent sitting at the edge of the mat with both arms elevated as well as independent standing with a standard walker (Gorgey and Gouda, 2022). Another follow-up study demonstrated the effects of percutaneous SCES implantation in two persons with chronic motor complete SCI (Gorgey et al., 2023a). In a C7 person with an American Spinal Cord Injury Impairment Scale (AIS) A, we noted that a configuration that elicited lower limb rhythmic activity (rhythmic-SCES) enhanced exoskeletal performance, and also facilitated trunk control (Gorgey et al., 2023a). In fact the rhythmic-SCES was also effective in reducing the extensor and flexor spasticity during different angular velocities (Gorgey et al., 2023b). Additionally, a T11 motor complete with an AIS B SCI was able to induce locomotor like stepping and attained overground stepping using a standard walker (Gorgey et al., 2023a).

Deciphering the lumbosacral rhythmicity configurations using a systematic approach may likely enhance several of the motor features and functional recovery in persons with SCI (Hofstoetter et al., 2021). In this case report, we showed that two interleaved rhythmic-SCES configurations induced specific motor adaptions when combined, rather than using each configuration separately, in an SCI person with T3 complete AIS A. Interleaved rhythmic-SCES configuration may provide more comprehensive activation of the locomotor circuitry than using a single mono-cathodal configuration.

This is a follow-up study to a T3 complete SCI who underwent SCES implantation in February 2022 in a specialized operating suite under fluoroscopy guidance at Louran Hospital in Alexandria, Egypt (Gorgey and Gouda, 2022). The implantation occurred approximately 4 years since SCI, which was caused by a motorcycle accident, and was subsequently followed by a spinal fusion that extended from T2 to T7 vertebrae. The participant signed research study consent forms for the surgical procedures, physiological assessments and activity-based training activities that were approved by the Hospital committee prior to enrollment in the trial. The entire procedure of implantation of the spinal cord stimulation device was conducted solely for research purposes. A detailed International Standards for Neurological Classification of SCI (ISNCSC) I exam was previously published for the participant (Gorgey and Gouda, 2022). A Medtronic Prime Advance Internal pulse generator with two 90 cm leads and eight contacts each were used. Briefly, the participant experienced migration of the left lead, after which and we configured the right lead to facilitate trunk control and standing using a standard walker (Gorgey and Gouda, 2022). The participant continued to work with his physical therapist to ensure enhancing his sit-to-stand performance using a standard walker. During this period, he was capable to achieve sit-to-stand activity for 3–5 min primarily using both arms and a standard walker while independently maintaining his extension of hips and knees without external assistance. In June 2022, the participant underwent a restorative surgery to recover the migrated lead. Three months later, x-ray was performed to verify the location of the leads as shown in Supplementary Figure S1A. Restoration of the left lead did not impact his ability to perform independent standing using a standard walker when the right lead was used for stimulating the spinal cord. Using previously published approach, we performed spatiotemporal mapping to assess the configurations that elicit activation of the rectus femoris (RF) followed by the medial gastrocnemius (m GM) muscles for the right lead (Day 1) and left lead (Day 2). The EMG relationship between RF and mGM was previously published (see Figure 5 in Gorgey and Gouda, 2022), highlighting the preceding EMG activities of RF muscle to mGM. All configurations were tested in a relaxed supine position using pulses adjusted at 2 Hz and 210 μs, the amplitude was progressed at an increment of 2 volts (Gorgey and Gouda, 2022). These resulted in five configurations that appear to meet the criteria of eliciting RF activities followed by mGM (Supplementary Figure S2). The selected configurations were sought to activate the full range of leg muscles to facilitate walking rather than only the extensor musculature in selective and non-selective manner (Hofstoetter et al., 2021).

**Figure 1:**
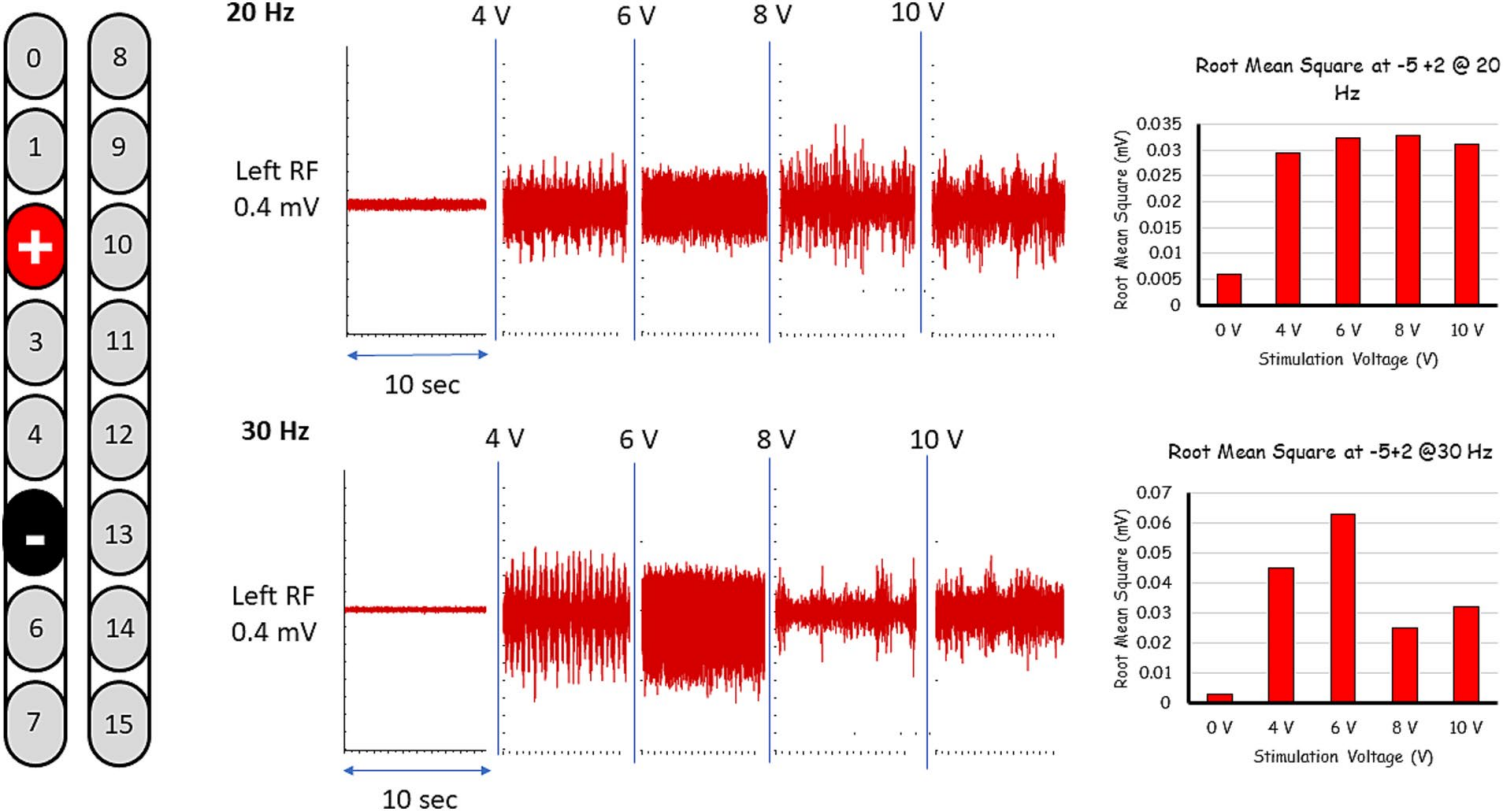
Representative rhythmic-SCES EMGs activities of the left rectus femoris (RF) muscle at 4-10 volts both at 20 and 30 Hz following setting the configuration at +2, –5 in supine lying position. The EMG traces are presented in both raw data (red). The EMG traces are presented as raw data. Root mean square (RMS) was used to quantify the amplitude of the muscle activity over 10 s intervals for each stimulation voltage, the resulting averages are presented in bar-graph format. L, left; RF, rectus femoris; mV, millivolts, Hz, hertz; sec, seconds.

**Figure 2:**
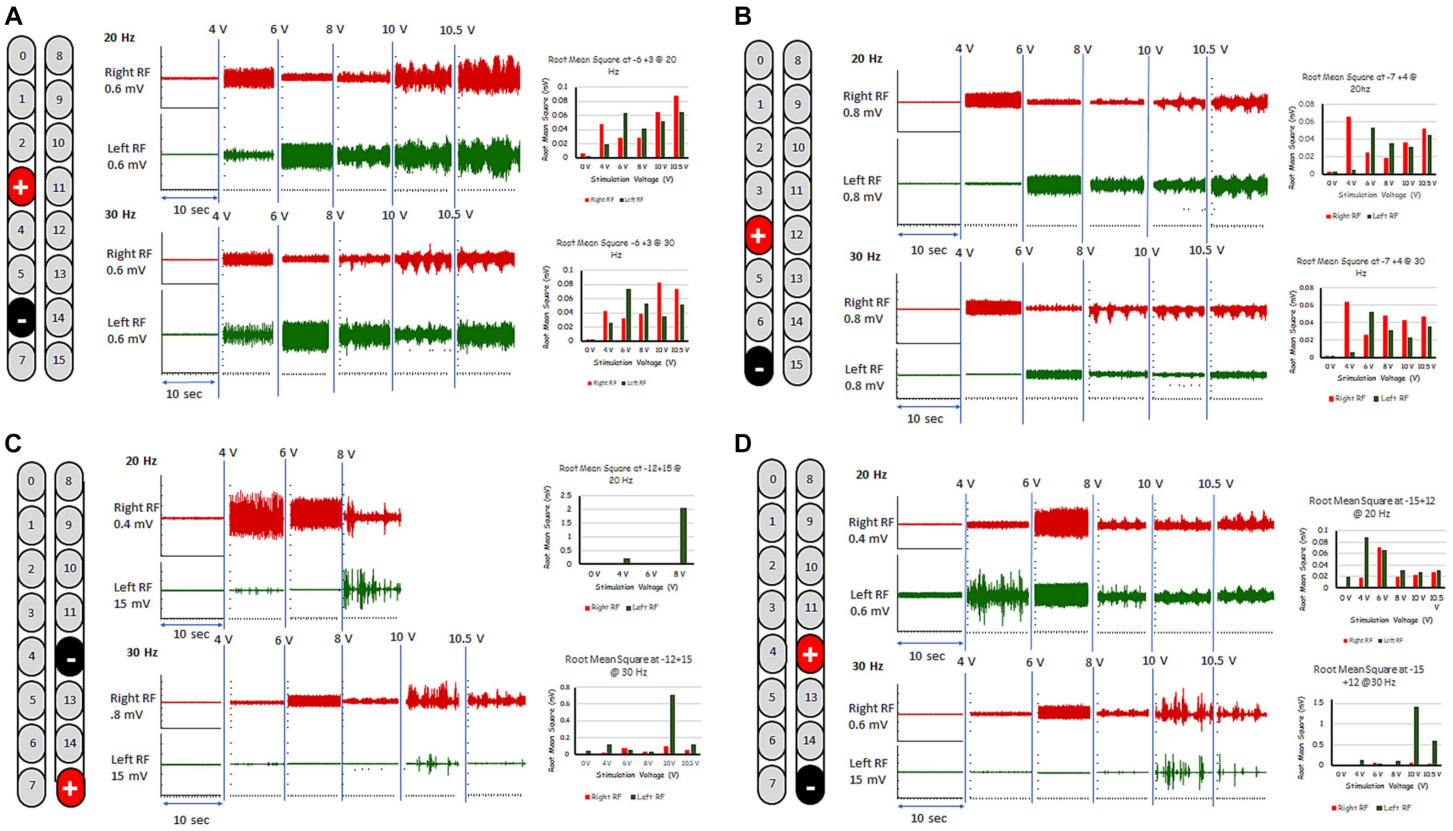
Rhythmic-SCES EMGs activities of the right and left rectus femoris (RF) muscle delivered in supine lying position at various amplitudes (4–10.5 volts) both at 20 and 30 Hz using multiple SCES configurations [**A** (+3, −6); **B** (+4, −7); **C** (−12, +15); **D** (+12, −15)]. Root mean square (RMS) was used to quantify the amplitude of the muscle activity over 10 s intervals for each stimulation voltage across configurations, the resulting averages are presented in bar-graph format. mV, millivolts; Hz, hertz; μs, sec, seconds.

These five configurations were further tested for spontaneous lower limb rhythmic activity in supine lying position using frequencies between 20 and 30 Hz (Figures 1, 2; see Supplementary Video S1). Representative raw and root mean square (RMS) EMG data of the electrode configuration “+2 and −5” is presented in Figure 1. In supine lying position, the EMG activities of the rhythmic-SCES configurations of the left and right RF muscles are presented (Figure 2). The five supine SCES-rhythmic configurations shared similar EMG activities when the amplitude progressed from 4 volts (spontaneous twitches on both knee extensors), 6 volts (sustained bilateral EMG activities with visible contraction of the knee extensors muscles), 8 volts (alternating visible activities between the right and left legs characterized by flexion of one leg and maintaining extension in the opposite leg.) and 10–10.5 volts (clear EMG oscillations accompanied with strong bursting without inducing alternating or reciprocal pattern).

**Figure 3:**
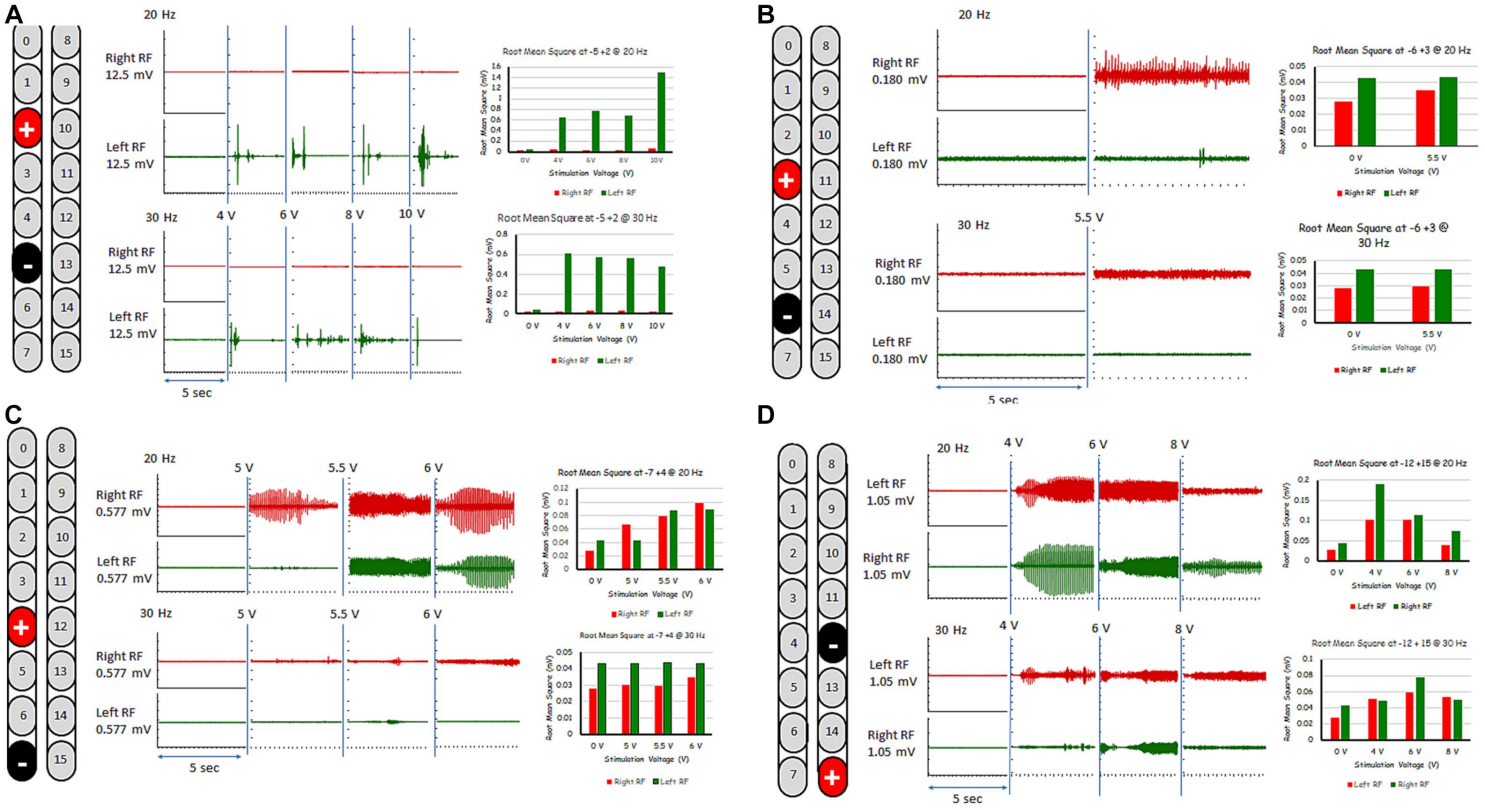
Rhythmic-SCES EMGs activities of the right and left rectus femoris (RF) muscles delivered in standing position between the parallel bars at various amplitudes (4–10 volts) both at 20 and 30 Hz using multiple SCES configurations [**A** (+2, −5); **B** (+3, −6); **C** (+4, −7); **D** (−12, +15)]. Root mean square (RMS) was used to quantify the amplitude of the muscle activity over 5 s intervals for each stimulation voltage across configurations, the resulting averages are presented in bar-graph format. mV, millivolts; Hz, hertz; μs, sec, seconds.

The five rhythmic-SCES configurations were further tested in an upright standing position between the parallel bars using either 20 or 30 Hz at a pulse duration of 210 μs (Figure 3). Table 1 highlighted the effects of these five configurations when tested in parallel bars at either 20 or 30 Hz. Before the testing began, the participant’s resting blood pressure was 117/60 mmHg. Participant was provided with two attempts to volitionally attempt to step for each configuration at an amplitude that started at 4 volts and then gradually progressed at 0.5 increments with pulses adjusted at 210 μs. Pulse duration was adjusted to 240 μs only when the configuration appeared to initiate upright standing and/or stepping in one of the legs.

**Table 1:** Testing the supine rhythmic-SCES configurations in a standing position between parallel bars at 20 and 30 Hz using a pulse duration of 210 μs.

Electrode configuration	Stim. frequency: 20 Hz	Stim. frequency: 30 Hz	Conclusion
+2, −5	Maintained upright position Attempted to step with right leg at 9.5 volts Strong bilateral knee flexion at 10 volts	One step with the right leg after the second attempt	Potentially can be used for step retraining
+3, –6	Failed to maintain upright standing Strong bilateral knee flexion Strong abdominal contraction prevented hip extension	Failed to maintain upright standing Started at 4 volts progressed to 5 volts Strong flexion synergy	Configurations were not appropriate for step retraining
+4, −7	Sit to stand required mild assistance for hip extension and locking the knees Trunk and hip assistance in upright position helped to overcome strong abdominal contractions Maintained upright standing position without external assistance Patient attempted one step	Strong flexion contraction of the abdominal muscles Strong bilateral knee flexion synergy Participant could not maintain upright position	Configurations were not appropriate for step retraining
−12, +15	Maintained standing upright position at 6.00 and 6.5 volts Strong hip adductors muscles manifested by crossing of the limbs Unfused tetanus of both knee extensors and unable to completely lock knees into full extension Managed to perform 3 steps only with the left leg at 8.00 or 8.5 volts	Unfused tetanus disappeared Unable to perform full back or trunk extension Participant had difficulty maintaining upright position or stepping when pulse duration is adjusted to 240 μs Participant maintained upright position and initiate left leg stepping at 8 volts when pulse duration is adjusted to 270 μs	Potentially can be used for step retraining
+12, –15	Configurations were not tested because conflict with other clinical procedures		

For the first 6 months, the “−5, +2” electrode configuration was the most effective to enhance stepping between parallel bars with and without external assistance; this task was practiced 2–3 times per week. The −5, +2 configuration elicited the most rhythmic muscle activity pattern especially at 6 volts and 30 Hz as highlighted in Figure 3. Step retraining started at a stimulation amplitude of 6 volts and was focused on the attempt to perform stepping for the left leg while weightbearing on the right leg, and vice versa. Manual assistance by trainers was provided to ensure appropriate stepping.

Within few days, the participant was able to achieve stepping between parallel bars with appropriate cueing from 2 assistants by spotting him from the back as well as to facilitate the leg movement. Additionally, the participant performed body weight supported treadmill (BWSTT) training to facilitate hip/knee flexion during stepping. The harness lifted approximately 10–15% of his total body weight during stepping at a speed of 1 km/h. Support increased up to approximately 30% of the body weight, if needed, to overcome extensor tone while performing BWSTT and the stimulation amplitude dropped to 3.5 volts.

Within 2 weeks, participant was able to achieve full weightbearing in upright standing position and shift his bodyweight between the left and right leg, using the upper limbs to assist with balance control. The therapist placed his foot between the participant’s legs to prevent leg crossing and ground friction during stepping. At this stage, stepping movement was initiated from the hip joint without achieving knee flexion.

Within 45 days from the beginning of practice with this spinal cord neuromodulation setup, the participant was capable of stepping through the entire 10 feet parallel bars with minimal support from the therapist, who placed his foot in between the participant’s legs to separate them and provided facilitation to the desired limb to advance forward. The stimulation amplitude used to facilitate stepping was as low as 5.7 volts (Supplementary Video S2).

Approximately 60 days from the beginning of practice, a commercially available “thigh master” was applied to the participant to mitigate lower limb adduction and crossing during stepping. This approach allowed the participant to step without the need of receiving any trainer’s assistance to avoid the crossing from both limbs while walking (Supplementary Video S2). The thigh master is essentially comprised of two metal tubes bent in a loop and connected and connected together.

In approximately 80 days, the participant started to practice overground stepping using a commercially available walker while still using the thigh master. The patient was successful in maintaining full upright standing position; however, he could not initiate volitional stepping as he was doing in the parallel bars. Nevertheless, after approximately one more month, the participant was capable of lifting his leg and initiate volitional stepping, covering the entire 10 feet distance of the parallel bar in less than 60 s. He kept using the thigh master, and the therapist provided minimal assistance during walking.

**Figure 4:**
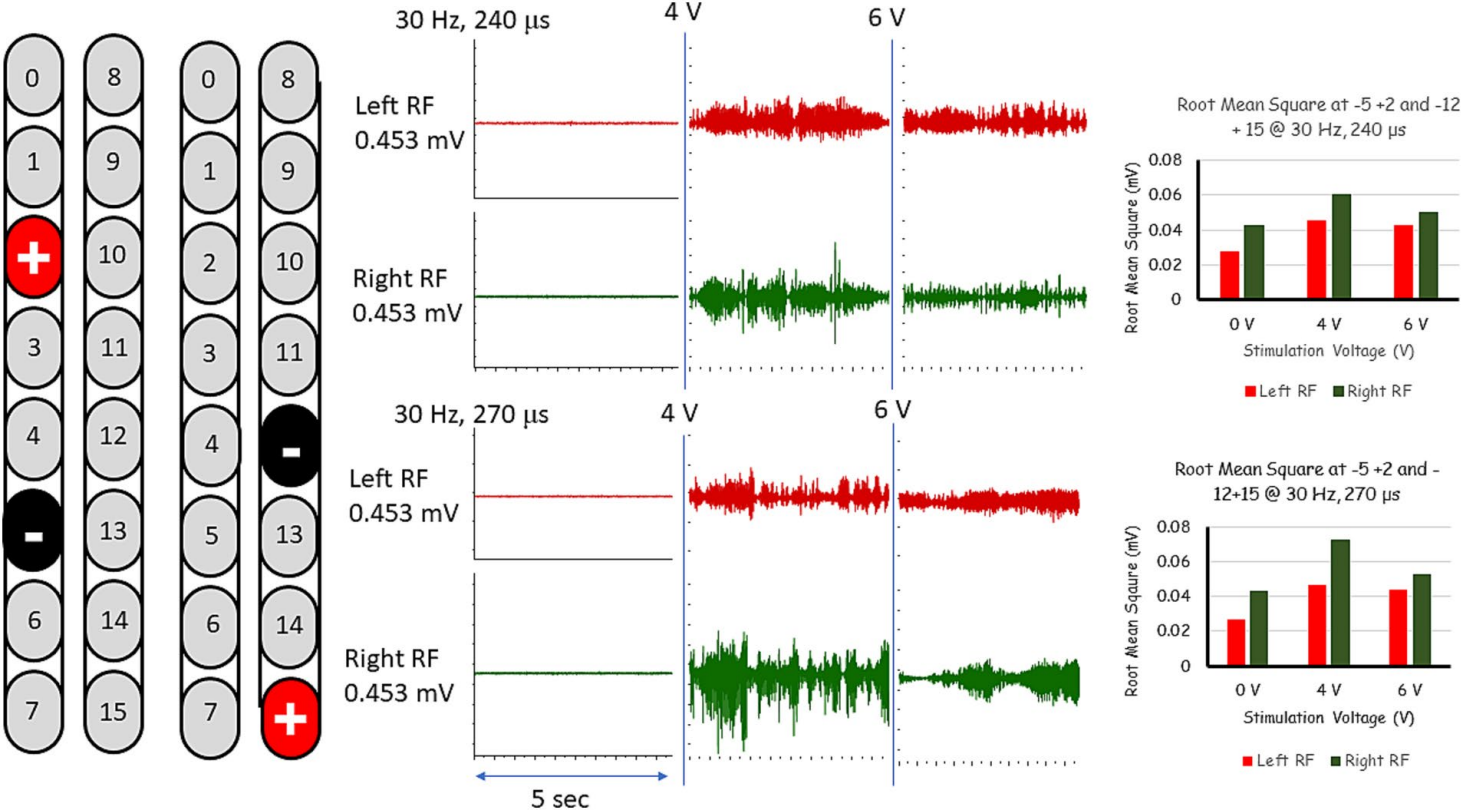
EMG activities following interleaved rhythmic-SCES configurations of the right and left rectus femoris (RF) muscles delivered in standing position between the parallel bars at various amplitudes (4 or 6 volts) and at pulse durations of either 240 or 270 μs. Root mean square (RMS) was used to quantify the amplitude of the muscle activity over 5 s intervals for each stimulation voltage, the resulting averages are presented in bar-graph format. mV, millivolts; Hz, hertz; μs, microseconds; sec, seconds.

After less than 6 months from the beginning of practice, we added a second stimulation program (electrode configuration: −12, +15; see Table 1) that was delivered in an interleaved fashion with the original program with the goal of reducing adductor tone and enhancing overground stepping. This was enough to remove the thigh master for all subsequent sessions of stepping between parallel bars and with overground stepping using a standard walker. The stimulation was set at 30 Hz, 240–270 μs and amplitude of 0.9–1.1 volts (Figure 4). The stimulation pulses of the two interleaved SCES programs were delivered with a 2 ms delay between them. We have also used Thera bands for both feet to provide dynamic support during stepping. Within a week, the participant was able to step independently, reciprocally advancing the right and left leg using a standard walker. After few months, participant was capable to perform 16 steps overground using a standard walker (Supplementary Video S3). Today, the participant is capable of performing 32 steps using the same training protocol.

## Discussion

In this case report, we have described activity-based training progression and a step-by-step procedure on how to select and manipulate percutaneous SCES configurations to achieve overground stepping in a person with a T3 complete paraplegia. In the first 5 months, with one SCES program resulting in unilateral stimulation, motor function of both lower limbs was facilitated and the participant was capable of achieving stepping between parallel bars; however, he could not initiate overground stepping with a walker. After a second SCES program was added on the opposite lead and was interleaved with the original program, the participant managed to perform overground stepping using a standard walker for approximately 16 steps as well as with a hydraulic lift that supported approximately 20–30% of his body weight during stepping. The rationale for interleaving with the second SCES-rhythmic configuration was based on providing more comprehensive activation of the locomotor circuitry. Furthermore, interleaving may allow more spatial awareness and motor guidance, which is likely to enhance inter-limb coordination between the left and right lower extremities during stepping.

We have reported successful stepping recovery in a person with a T11 AIS B using SCES delivered by percutaneous leads (Gorgey et al., 2023a). It is possible to assume that engagement of trunk muscle may have facilitated stepping in a person with a T11 injury. However, it is not the case in a person with T3 AIS A. In both reports, the stepping pattern did not demonstrate clear knee flexion during the swing phase (Gorgey et al., 2023a). The exact underlying mechanism related to this stepping pattern is yet to be determined. However, we believe that training stimuli may have been inefficient to facilitate hip/knee flexion during stepping. We may need to alter our training paradigm to eventually train hip/knee flexion in lying position before transitioning to standing-stepping activities. Surprisingly, in our earlier report, the T11 participant could successfully initiate hip/knee from both side and supine lying position (Gorgey et al., 2023a). However, he failed to translate this flexion pattern into overground stepping. We assumed that the strong extensor tone overrode the flexion synergy during stepping (Gorgey et al., 2023a). The participant initially relied on hip hiking and rotation of the pelvis to facilitate stepping (bracing of the leg); albeit he could induce up to 16 steps with a standard walker.

Historically, the recovery of standing and stepping was not possible for persons with complete SCI. Several other research groups successfully demonstrated that neuromodulation or targeted stimulation of the lumbosacral segments can restore overground mobility in persons with complete and incomplete SCI (Harkema et al., 2011; Angeli et al., 2018; Wagner et al., 2018; Gorgey et al., 2020). The use of percutaneous SCES successfully transforms the excitability of dormant spinal locomotor centers into more active neural circuitries via integration of the spared descending supraspinal motor signals with the afferent proprioceptive input (Taccola et al., 2020; Gill et al., 2021; Hachmann et al., 2021). This neuromodulation approach together with several months of overground training successfully facilitated stepping-like activity and the ability to intentionally initiate overground mobility. We demonstrated that less invasive percutaneous SCES may eventually lead to comparable results and even in participant who may disqualify from paddle implantation (Gorgey et al., 2023a).

The use of SCES rhythmic configuration targeted toward stepping resulted in both standing and stepping. Previous work noted that separate configurations were needed to achieve standing separately from stepping (Rejc et al., 2015). The authors suggested that training the spinal cord circuitries to exclusively achieve stepping may hinder the ability to stand in complete SCI (Rejc et al., 2017). Therefore, practicing both standing and stepping paradigms may promote improvements in both motor tasks. The current findings are in line with recent findings that showed that rhythmic SCES configurations reduced spasticity and enhanced motor function in a person with complete tetraplegia (Gorgey et al., 2023b). Similarly, participant’s exoskeletal performance was also shown to improve with SCES on compared to stepping with SCES off (Gorgey et al., 2023a).

The neurorehabilitation program reported in the current report gradually enhanced stepping between the parallel bars via enhancing pelvic hiking and stepping without clear engagement of the hip flexor muscles. Surprisingly, the participant was capable of partially initiating hip flexion in supine lying position; however, translation of this motor behavior in standing could not happen, similarly to our earlier findings (Gorgey et al., 2023a). The additional weightbearing-related sensory information conveyed to the spinal circuitries predominantly favored extensor synergy and overriding any flexion pattern (Harkema et al., 2011). Modulating this extensor tone during the swing phase may be facilitated by additional stimulation specifically oriented toward engaging the flexor withdrawal behavior or using external electrical stimulation of the TA muscle or the common peroneal nerve that may facilitate dorsiflexion accompanied with hip flexion. It is likely to assume that trunk muscles were partially engaged together with knee extensors to facilitate stepping without clear hip flexion.

The implemented training program relied on progressively allowing the participant to enhance his neuroplasticity capabilities via integrating SCES with locomotor training. The program was limited only 2–3 times per week for 2 h per session. It is empirical to note that motor gains did not plateau as demonstrated by his ability to increase the number of overground stepping up to 16 steps. Others have noted a plateau in motor recovery after SCES implantation and intensive rehabilitation (Lorach et al., 2023). This is likely to assume that additional neural pathways may likely to recover and promote additional functional gains with rehabilitation. The use of interleaved program represents an important advantage compared with the use of a single program, because it facilitates access to different spinal cord circuitries with different voltages that are responsible for stepping (Rejc and Angeli, 2019). The control of locomotion stepping is derived via multiple pathways and engaging multiple pathways would result in composite pattern to enhance the motor skills responsible for stepping. It is feasible to assume that the addition of second SCES-rhythmic configuration may engage more circuitries responsible for overground locomotion (Gerasimenko et al., 2008). This resulted in more comprehensive activation of different spinal cord circuitries than using a single configuration. The current findings are in accordance with previous findings that noted improvement in overground locomotion following applications of interleaved configurations compared to single configuration (Gill et al., 2018). Future studies are warranted to better understand the mechanisms behind interleaved configurations. Furthermore, the two parallel percutaneous leads provided lateral activation of each lead. Future trials should consider interleaving the same lead with respective timing for each configuration.

The current overreaching goal is to transition from supervised training program to a daily home-base program to facilitate overground stepping to the bathroom or the kitchen. Daily stepping may provide more functional goals for the participant via incorporating SCES into activities of daily living. The participant used the SCES system over the last 12 months and became more familiar with using his own remote control to access the implanted SCES and turn it on and off as well as ramping up or down the stimulation amplitude. This ability to safely control the SCES is critical to independently use in his daily routine. Therefore, exploring home-use applications is considered an important future step for persons with SCI.

In summary, activity-based training with two rhythmic interleaved percutaneous SCES configurations reduced adductor crossing muscle tone and enhanced overground stepping with a standard walker in a person with complete paraplegia. The two SCES interleaved programs may have enhanced inter-limb coordination via spatial control of left and right lower extremities. The participant demonstrated the ability to perform stepping-like patterns without hip/knee flexion only with SCES on. The current outcomes support our earlier findings that percutaneous SCES implantation may serve as an alternative rehabilitation tool for restoration of overground mobility in persons with complete SCI.

## Data availability statement

The raw data supporting the conclusions of this article will be made available by the authors, without undue reservation.

## Ethics statement

Written informed consent was obtained from the individual(s) for the publication of any potentially identifiable images or data included in this article. Written informed consent was obtained from the participant for publication of the case report.

## Author contributions

AG: Conceptualization, Data curation, Formal analysis, Investigation, Methodology, Project administration, Supervision, Writing – original draft, Writing – review & editing. SV: Formal analysis, Methodology, Writing – review & editing. MR: Writing – review & editing. BG: Project administration, Resources, Supervision, Writing – review & editing. ER: Formal analysis, Methodology, Writing – original draft, Writing – review & editing. JG: Conceptualization, Data curation, Investigation, Writing – original draft, Writing – review & editing.

## Supplementary material

SUPPLEMENTARY FIGURE 1 x-ray images demonstrated position of the two SCES percutaneous leads at different timepoints after implantation. **a**) x-ray antero-posterior view was captured 3 months after restoration of the SCES leads to cover mid T11-mid L1. **b**) x-ray antero-posterior view that was captured 150 days post-the original image. The x-ray images demonstrated that SCES leads maintained their position and did not experience migration.

SUPPLEMENTARY FIGURE 2 The five supine SCES-rhythmic configurations that were tested in supine lying position based on EMG activities that turned on the rectus femoris muscles followed by medial gastrocnemius muscle activities at 2 Hz (Gorgey and Gouda, 2022). The five configurations were selected out of 20 configurations that covered both SCES leads using a systematic approach that was previously highlighted by our group (Gorgey and Gouda, 2022).

SUPPLEMENTARY VIDEO 1 Representative rhythmic oscillations pattern elicited at supine lying position using +12, -15 configuration adjusted at 30 Hz.

SUPPLEMENTARY VIDEO 2 Using the thigh master to facilitate stepping between parallel bars using a single configuration of -2, +5 that was set at 6 volts, 30 Hz and 240 µs. Note the participant with maintaining his legs apart managed to easily cover the 10-feet distance of the parallel bars using both arms without external assistance from the therapist.

SUPPLEMENTARY VIDEO 3 using interleaved configurations of -2, +5 and -12 +15 to perform overground stepping. Note elastic straps were provided to support the feet and reduce friction against the floor during stepping. The participant initially required the therapist to support the walker during stepping and another assistant to provide spotting without any assistance. Few months after using interleaved configurations, participant performed up to 16 overground steps without any external support from therapist while using a standard walking.
