# Supplementary figures and images for: Interleaved configurations of percutaneous epidural stimulation enhanced overground stepping in a person with chronic paraplegia
Source: Front Neurosci. 2023 Dec 7;17:1284581. doi: 10.3389/fnins.2023.1284581 (PMC10740173; doi:10.3389/fnins.2023.1284581)

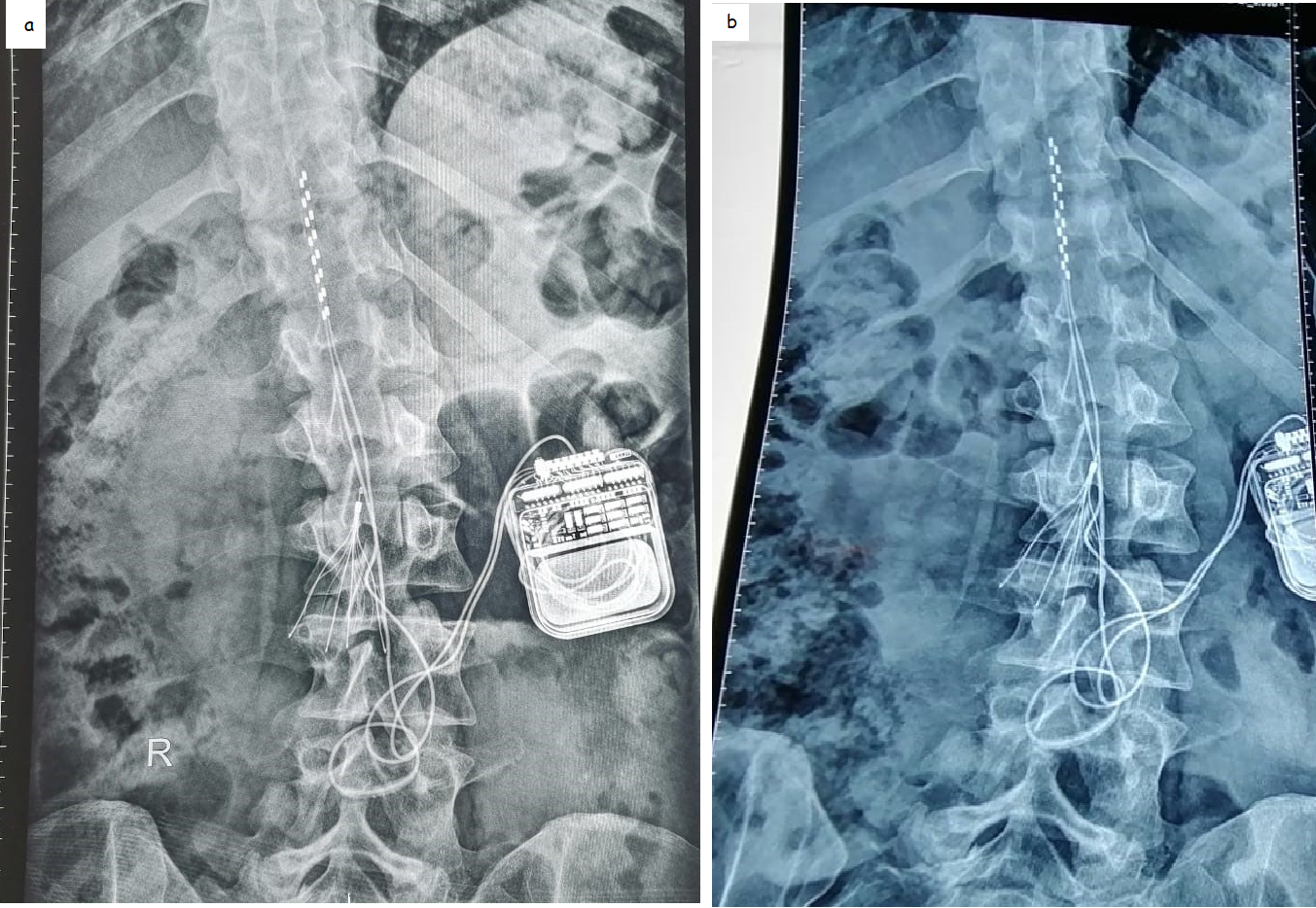

Supplement: SUPPLEMENTARY FIGURE 1 — x-ray images demonstrated position of the two SCES percutaneous leads at different timepoints after implantation. a) x-ray antero-posterior view was captured 3 months after restoration of the SCES leads to cover mid T11-mid L1. b) x-ray antero-posterior view that was captured 150 days post-the original image. The x-ray images demonstrated that SCES leads maintained their position and did not experience migration. [file Image_1.TIF]

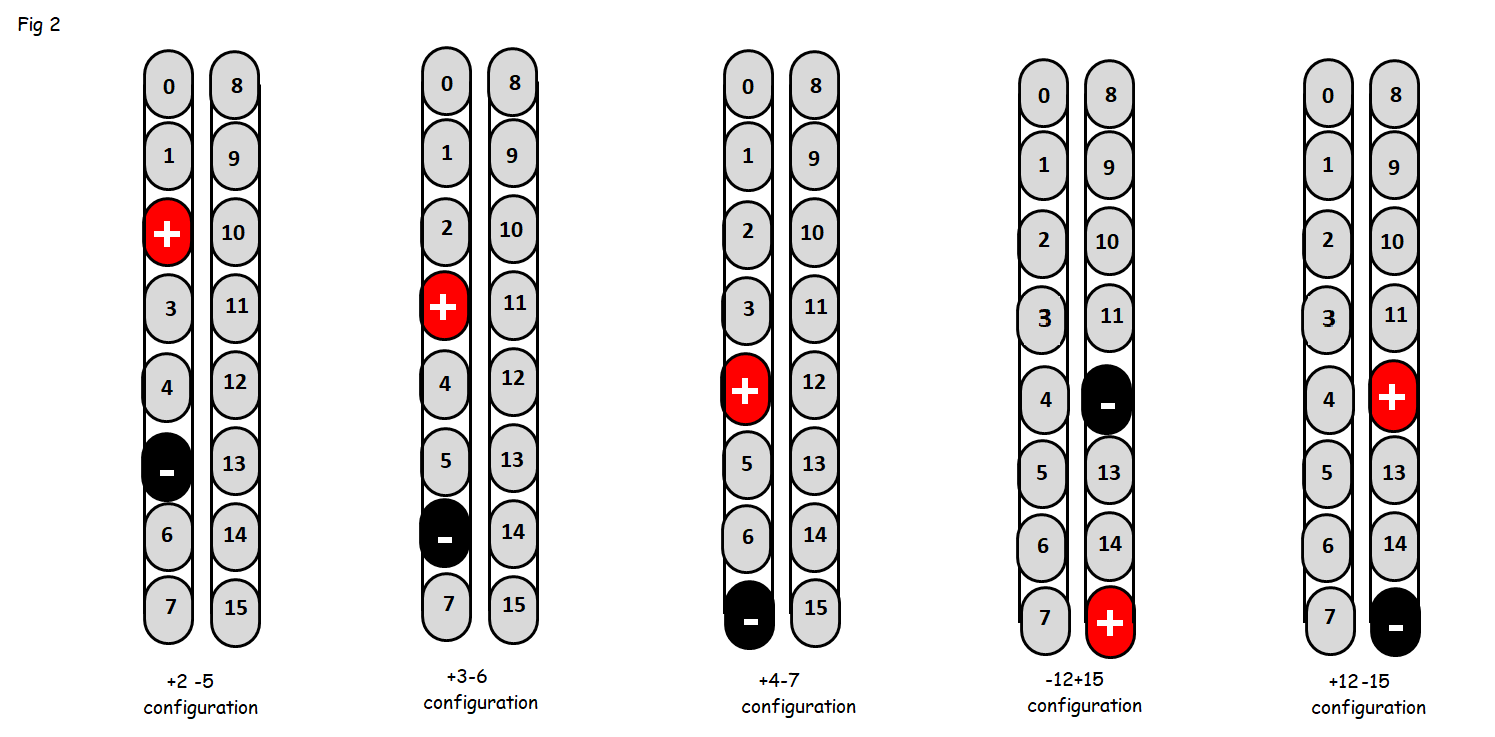

Supplement: SUPPLEMENTARY FIGURE 2 — The five supine SCES-rhythmic configurations that were tested in supine lying position based on EMG activities that turned on the rectus femoris muscles followed by medial gastrocnemius muscle activities at 2 Hz (Gorgey and Gouda, 2022). The five configurations were selected out of 20 configurations that covered both SCES leads using a systematic approach that was previously highlighted by our group (Gorgey and Gouda, 2022). [file Image_2.TIF]
