# Supplementary material for: Interleaved configurations of percutaneous epidural stimulation enhanced overground stepping in a person with chronic paraplegia
Source: Front Neurosci. 2023 Dec 7;17:1284581. doi: 10.3389/fnins.2023.1284581 (PMC10740173; doi:10.3389/fnins.2023.1284581)
Supplement: Supplementary file 6 [file Presentation_1.pdf]

Patient Perspective

I participated in this program since February 2022. During this time, I noticed several gains including my ability to secure sitting at the edge of the mat and independently secure standing with a standard walker. I realized that I am still using my arms to achieve these important functions. However, these functions were not even possible before the implantation. Most recently with the help of the team, I managed to achieve stepping between parallel bars and then stepping overground with using the standard walker. I understand that my walking pattern is far from normal but, with training and remapping of the leads, I am on the path to achieve this pattern with hips and knees flexion.
